# Single-Dose Intranasal Immunization with ChAd68-Vectored Prefusion F Vaccines Confers Sustained Protection Against Respiratory Syncytial Virus in Murine Models

**DOI:** 10.3390/vaccines13050528

**Published:** 2025-05-15

**Authors:** Jing Miao, Xuejie Li, Yingwen Li, Lingjing Mao, Wenkai Suo, Jiaming Lan

**Affiliations:** 1 Shanghai Institute of Immunity and Infection, Chinese Academy of Sciences, Shanghai 200031, China; jmiao@siii.cas.cn (J.M.);; 2 University of Chinese Academy of Sciences, Beijing 100049, China

**Keywords:** respiratory syncytial virus, chimpanzee adenoviral vector, pre-fusion conformation, vaccine, immune responses, long-term protection

## Abstract

**Background/Objectives:** Respiratory syncytial virus (RSV) poses a substantial global health threat, particularly impacting infants and vulnerable pediatric populations through severe respiratory morbidity. **Methods:** We developed a novel adenoviral vector vaccine platform utilizing chimpanzee adenovirus 68 (AdC68) to deliver prefusion F (pre-F) antigens from RSV subtypes A and B, generating three vaccine candidates: AdC68-A (subtype A), AdC68-B (subtype B), and AdC68-A+B (bivalent formulation). **Results:** Single intranasal (i.n.) immunization and prime–boost immunizations via intramuscular (i.m.) routes in BALB/c mice induced robust immune activation, with single i.n. administration conferring durable protection evidenced by an 85% reduction in pulmonary viral loads (*p* < 0.05) at 134 days post-immunization. All vaccine formulations via i.n. single administration elicited potent subtype-specific IgG responses (geometric mean titers 50–12,800) and Th1-polarized cellular immunity (552–1201 IFN-γ+ spot-forming units/10^6^ PBMCs, IgG2a/IgG1 > 1) in bivalent formulation group, while i.m. boosting enhanced cellular responses 3-fold versus prime immunization alone (*p* < 0.01). Notably, despite undetectable serum-neutralizing antibodies and absent mucosal IgA in bronchoalveolar lavage at 7 days post-i.n. immunization, the sustained viral control highlights non-neutralizing antibody-mediated protective mechanisms. **Conclusions:** These findings establish the proof-of-concept for adenoviral-vectored intranasal vaccines against RSV, though optimization of humoral response induction and mucosal immunity duration require further investigation.

## 1. Introduction

The respiratory syncytial virus (RSV), first isolated from chimpanzees with respiratory illness in 1956 [1], evolved into a globally prevalent pathogen imposing substantial disease burdens [2,3]. While RSV infections in immunocompetent adults typically manifest as mild upper respiratory symptoms, they represent the predominant cause of severe lower respiratory tract infections (LRTIs) in infants, and also are clinically significant agents of pneumonia in older adults [3,4,5,6,7,8], frequently progressing to acute bronchiolitis and apnea in vulnerable populations [9,10]. Epidemiological studies estimate that over 90% of children experience primary RSV infection before age two, with recurrent infections persisting throughout adulthood [11]. Globally, RSV is responsible for approximately 33 million annual cases of acute LRTIs in children under five years old, culminating in nearly 60,000 pediatric deaths [12,13,14]. Concurrently, older adults face 3–10% annual infection rates, with severe cases requiring hospitalization occurring in 0.1% of this demographic [15].

This substantial disease burden underscores the urgent need for effective prophylactic interventions. Vaccine development has consequently emerged as a global health priority [8,16,17]. Current investigational strategies encompass diverse platforms including live-attenuated vaccines, subunit formulations, mRNA-based candidates, recombinant viral vectors, and nanoparticle vaccines [2,18,19]. Among these, replication-deficient adenoviral vectors demonstrate particular promise due to their genomic stability, high antigen-loading capacity, broad tissue tropism, and established safety profile [20,21,22,23,24].

The RSV genome encodes 11 proteins through 10 genes, including eight structural components [2,13]. Three transmembrane glycoproteins—small hydrophobic (SH), attachment (G), and fusion (F) proteins—mediate viral entry, with the G protein facilitating cellular attachment and the F protein driving membrane fusion [2,25,26]. These surface glycoproteins constitute primary vaccine targets. Notably, sequence variation in G protein enables classification into RSV-A and RSV-B subtypes that typically co-circulate with alternating predominance [27,28].

While both F and G proteins elicit neutralizing antibodies, the F protein’s superior immunogenicity stems from multiple antigenic sites and greater conservation across RSV subtypes [29,30]. This molecular stability positions the F protein as the preferred target for vaccine design. Structural analyses reveal two conformational states: a metastable pre-fusion (pre-F) form mediating viral entry, and a stable post-fusion (post-F) trimer formed post-membrane fusion [2]. Crucially, pre-F conformation induces more potent neutralizing antibodies than its post-F counterpart [16,19,31,32,33]. This immunogenic superiority underpins the design of three recently FDA-approved RSV vaccines (Arexvy, ABRYSVO, and mRNA-1345), all targeting pre-F conformation [14,17].

In this study, we developed three replication-deficient chimpanzee adenovirus (AdC68) vectored vaccines expressing RSV-A pre-F (AdC68-A), RSV-B pre-F (AdC68-B), or both antigens (AdC68-A+B). Through a prime–boost immunization regimen combining intramuscular and intranasal administration in murine models, we systematically evaluated the vaccines’ immunogenicity and protective efficacy against the RSV challenge.

## 2. Materials and Methods

### 2.1. Ethics Statement

All procedures involving RSV and animal subjects were conducted in accordance with Biosafety Level 2 (BSL-2) standards at the Shanghai Institute of Immunity and Infection, Chinese Academy of Sciences (Approval number: P2024028). Strict adherence to the ethical guidelines governing animal experimentation and research was maintained throughout the study.

### 2.2. Cells and Viruses

HEK293 cells (CRL-1573) and Hep-2 cells (CCL-23) were maintained in high-glucose Dulbecco’s Modified Eagle Medium (DMEM; Invitrogen, Carlsbad, CA, USA) containing 10% heat-inactivated fetal bovine serum (FBS; Invitrogen, CA, USA), 100 U/mL penicillin, and 100 µg/mL streptomycin. Cell cultures were incubated under standard conditions (37 °C, 5% CO_2_, humidified atmosphere). The RSV long strain was propagated in Hep-2 cell monolayers, with viral titers subsequently determined by plaque formation assay following established protocols. Mycoplasma contamination was rigorously excluded from all cell lines.

### 2.3. Production of AdC68-Based RSV Vaccines

The full-length F genes of RSV-A and RSV-B subtypes were retrieved from the GenBank database of the National Center for Biotechnology Information (NCBI) [http://www.ncbi.nlm.nih.gov/]. Sequence alignment was performed using MEGA Version 11.0.13 to identify conserved regions, leading to the selection of F gene sequences from RSV-A (Accession number: MZ221194.1) and RSV-B (accession number: MF445853.1) as reference sequences. Subsequent modifications included deletion of the P27 sequence and two Furin cleavage sites, replaced by a GS linker (GS). Unnatural disulfide bonds (S155C and S290C) and hydrophobic cavity-filling mutations (S190F and V207L) were introduced into the F protein, effectively locking in the pre-F conformation and preventing its transition to post-fusion (post-F) [34], and the cytoplasmic domain was removed. The refined RSV-A/B pre-F sequences were codon-optimized and inserted into a pShuttle2 vector between the NotI and KpnI restriction sites by GenScript (Nanjing, China).

The pre-F expression cassette containing the CMV promoter, pre-F gene, and polyadenylation (poly-A) signal from the simian virus 40 (SV40) tail was excised using I-CeuI and PI-SceI restriction enzymes (NEB, Ipswich, MA, USA) and inserted into the E1-deleted region of the AdC68 plasmid to generate the recombinant plasmids pAdC68-A Pre-F, pAdC68-B Pre-F, and pAdC68-A+B Pre-F. Empty pAdC68 was used as a sham control without insertion at the E1-deleted locus. The constructed plasmids were linearized with PacI and transfected into HEK293 cells to rescue AdC68-A, AdC68-B, and AdC68-A+B. The recombinant adenoviruses AdC68-A, AdC68-B, AdC68-A+B, and empty control AdC68 were propagated and purified by cesium chloride density gradient centrifugation, and viral particles were quantified using spectrophotometry. After confirming the correct clones using restriction enzyme digestion, the recombinant adenovirus vaccines were stored at −80 °C.

### 2.4. The Cytopathic Effect (CPE) Observation and Western Blot Analysis

HEK293 cells cultured in 6-well tissue culture plates (NEST, Wuxi, China) were infected with recombinant adenoviruses AdC68-A, AdC68-B, AdC68-A+B, and AdC68, respectively. Twenty-four hours post-infection, CPE was observed under an Olympus IX73 inverted microscope (Olympus, Tokyo, Japan), and images were captured. Cell supernatants were harvested when CPE no longer progressed and mixed with 4× non-denaturing protein loading buffer (Solarbio, Beijing, China; Cat No. P1017). Each cell lysate was separated by 10% SDS-polyacrylamide gel electrophoresis (SDS-PAGE) (Epizyme, Shanghai, China; Cat No. PG112) and transferred to a nitrocellulose (NC) membrane. NC membranes underwent blocking through 1 h incubation in TBST (Tris-buffered saline containing 0.1% Tween 20) supplemented with 5% non-fat dried milk. Primary detection employed the D25 monoclonal antibody (generously provided by Prof. Sandra Qiu, University of Science and Technology of China) targeting pre-fusion F protein conformations. Subsequent detection utilized a species-matched HRP-conjugated secondary antibody (Proteintech, Wuhan, China; Cat No. SA00001-17) applied at 1:10,000 dilution in a blocking buffer. Chemiluminescent signal development was achieved using Tanon™ High-Sig ECL Substrate (Tanon Science & Technology, Shanghai, China; Cat No. 180-5001) following manufacturer-recommended exposure parameters.

### 2.5. Visualization of Viral Particles by Negative Staining Electron Microscopy (EM)

Copper grids (EMCN, Beijing, China) were hydrophilically treated with the carbon film facing upward. A 10 µL sample was dropped onto the grid and allowed to stand for 60 s. The filter paper was used to absorb the solution vertically along the edge of the grid at an angle close to 90 degrees. Immediately, 10 µL of uranium acetate staining solution was added and allowed to stand for 60 s. The residual solution was blotted out with forceps, and the grids were dried and stored in a suitable container (grid box or petri dish). The grids were examined and photographed using a Field Emission Transmission Electron Microscope (Tecnai G2 F20 TWIN; FEI, Cincinnati, OH, USA).

### 2.6. Animals, Immunization, Challenge, and Sample Collection

Four- to six-week-old female BALB/c mice were purchased from Beijing Vital River Laboratory Animal Technology Co., Ltd. (Beijing, China) and maintained under specific pathogen-free conditions with food and water provided ad libitum. Mice were immunized intramuscularly (i.m.) or intranasally (i.n.) with 1 × 10^10^ viral particles (VPs) of the vaccines.

For i.m. immunization, mice were randomly divided into four groups and received two doses of immunization on day 0 and day 21. Groups were vaccinated with AdC68-A, AdC68-B, AdC68-A+B, or empty AdC68, respectively. On day 7 after each vaccination, four mice per group were sacrificed, and splenocytes were harvested to assess cellular immune responses. Sera samples were collected at the indicated time points to detect humoral immune responses (*n* = 5 per group).

For i.n. immunization, mice were randomly divided into four groups and received one dose of immunization. After anesthesia with isoflurane, mice were vaccinated with AdC68-A, AdC68-B, AdC68-A+B, or empty AdC68 at a dose of 1 × 10^10^ VPs. On day 7 post-immunization, three mice per group were sacrificed, and bronchoalveolar lavage (BAL) samples were collected to detect IgA, while splenocytes were harvested for cellular immune response analysis. Sera samples were collected at the indicated time points to detect humoral immune responses (*n* = 6 per group).

Subsequently, 42 or 131 days after the last immunization in the i.m. or i.n. groups, respectively, mice were anesthetized with isoflurane and challenged with 1 × 10^6^ plaque-forming units (PFUs) of RSV via endotracheal intubation using a trace liquid endotracheal nebulizer and laryngoscope. Animal weights were recorded daily. Three days post-challenge, mice were euthanized, and lung tissues were collected for viral load determination by qRT-PCR.

### 2.7. Antibody Detection by Enzyme-Linked Immunosorbent Assay (ELISA)

IgG-specific binding antibodies were assessed by ELISA. Immunosorbent plates (96-well format, Jet Bio-Fil, Guangzhou, China) were functionalized through overnight incubation (4 °C) with 100 ng/well of either: (a) recombinant pre-fusion stabilized F protein trimer (DS-Cav1), or (b) post-fusion conformation F protein (generously provided by Prof. Sandra Qiu, USTC), diluted in coating buffer (Solarbio, Beijing, China; Cat No. C1055). Following three successive washes with phosphate-buffered saline (PBS, pH 7.4), non-specific binding sites were saturated using 100 μL blocking solution containing 5% *w*/*v* non-fat dried milk in PBS (37 °C, 90 min).

Serial serum dilutions (initial 1:50 dilution, 4-fold increments) and bronchoalveolar lavage (BAL) samples were incubated with antigen-coated wells (37 °C, 2 h) under constant agitation. Post-incubation plates underwent four PBST (0.05% Tween-20 in PBS) washing cycles (2 min/wash) prior to 1 h incubation (37 °C) with species-specific HRP-conjugated immunoglobulins: anti-mouse IgG (Abcam, Cambridge, UK; Cat No.ab6789; 1:30k), IgA (Abcam, Cambridge, UK; Cat No. ab97235; 1:20k), IgG1 (Abcam, UK; Cat No.ab97240; 1:50k), or IgG2a (Abcam, Cambridge, UK; Cat No. ab97245; 1:50k) diluted in blocking matrix.

After final PBS rinsing, enzymatic activity was quantified through TMB chromogenic conversion (NCM Biotech, Suzhou, China; Cat No. M30500; 100 μL/well, 15 min dark incubation), terminated by acidification (50 μL stop solution, Solarbio, Beijing, China; Cat No.C1058). Optical density at 450 nm was quantified using a microplate reader (BioTek Synergy H1, BioTem, Winooski, VT, USA). Antibody endpoint titers were defined as the maximal dilution yielding OD450 values ≥ 2.1× negative control means.

### 2.8. Evaluation of Antigen-Specific T-Cell Responses by Enzyme-Linked Immunospot (ELISpot) Assay

The ELISpot assay was performed on isolated murine splenocytes using the ELISpot Plus: Mouse IFN-γ (ALP) kit (Mabtech, Nacka Strand, Sweden; Cat No. 33214APT-10) according to the manufacturer’s instructions. Briefly, plates were activated with PBS and blocked with RPMI 1640 medium (Invitrogen, CA, USA) containing 10% FBS for 30 min. Splenocytes were collected and seeded at 5 × 10^5^ cells per well, experimental cultures received RSV pre-F-specific overlapping peptide arrays (18-residue sequences with 10-amino acid offsets, 5 µg/mL individual peptide concentration) for T cell activation. Polyclonal activation controls employed a mitogen cocktail containing PMA (phorbol 12-myristate 13-acetate, 500 ng/mL) and calcium ionophore ionomycin (10 µg/mL; Dakewei Biotech, Shenzhen, China; Cat No. 2030421), while baseline measurements utilized mock-treated wells containing complete medium alone. Cellular responses were assessed following 20 h incubation under physiological culture conditions (37 °C, 5% CO_2_). IFN-γ spot-forming cells were detected using the detection antibody R4-6A2-biotin (1 µg/mL), followed by streptavidin-ALP (1:1000 dilution in PBS containing 0.5% FBS) for 1 h at room temperature. The spots were developed with ELISpot substrate BCIP/NBT-plus for ALP. The number of spots was counted using an ELISpot Reader (S6 Universal, CTL, Cleveland, OH, USA).

### 2.9. Neutralization Assay

Mouse serum samples were incubated at 56 °C for 30 min to ensure complete inactivation of the complement system prior to further analysis. Starting at a 1:20 dilution, heat-inactivated sera were serially diluted four-fold in DMEM/F12 (Invitrogen, CA, USA). Samples were mixed with 100 PFU of RSV and incubated at 37 °C for 1 h. The virus-serum mixtures were then incubated with Hep-2 cells in a 12-well plate (Corning, NY, USA) at 37 °C with 5% CO_2_ for 2 h. After incubation, the supernatant was removed, cells were washed with PBS, and 1.5 mL of DMEM/F12 medium containing 0.6% Avicel, 2% FBS, and 1% penicillin–streptomycin (PS) was added to each well. The plates were incubated at 37 °C for 72 h. After incubation, the medium was removed, cells were washed twice with PBS, and 300 µL of 4% paraformaldehyde fixing solution was added to each well. After fixing for 30 min at room temperature, the fixing solution was discarded, and 300 µL of 1% crystal violet staining solution was added. After staining for 20 min, the staining solution was gently washed off with running water. The number of plaques was counted after drying.

### 2.10. Viral Load Detection by Real-Time Quantitative Reverse Transcriptase PCR (qRT-PCR)

Mouse lung tissues were weighed and homogenized in 1 mL of DMEM using a tissue grinder with 10 steel balls (2 mm). Virus RNA was extracted from 100 µL of the homogenate supernatant using the Viral Genome DNA/RNA Extraction Kit (TIANGEN, Beijing, China; Cat No.DP315) according to the manufacturer’s instructions, and the total RNA was eluted in 20 µL of RNase-free water. RNA (1 µL) was used as the template for qRT-PCR. Viral RNA amplification reactions were conducted using the LightCycler 480 II system (Roche, Basel, Switzerland) with the HiScript^®^ II One-Step Probe RT-qPCR Master Mix (Vazyme Biotech, Nanjing, China; Cat No. Q223-01) specific to the RSV N nucleoprotein coding region. Oligonucleotide sequences included the following: forward primer 5′-ggcagtagagttgaagggattt-3′, reverse primer 5′-tgcacactagcatgtcctaac-3′, and dual-labeled TaqMan probe (FAM-5′-tatgaatgcctatggtgcagggca-3′-BHQ1). Quantitative calibration utilized an eight-point logarithmic standard series spanning 10^10^–10^3^ target copies per reaction (20 μL total volume). Viral loads were expressed as viral copies per milligram, calculated using the standard curve.

### 2.11. Statistical Analysis

Statistical analysis was performed using GraphPad Prism 9.0.0 software. Data are presented as mean ± SD. *p*-values were analyzed using two-tailed unpaired Student’s *t*-tests (****, *p* < 0.0001; ***, *p* < 0.001; **, *p* < 0.01; *, *p* < 0.05; ns, *p* ≥ 0.05)

## 3. Results

### 3.1. Successful Rescue of Recombinant Chimpanzee Adenovirus Expressing RSV-A and/or B pre-F Protein

**Figure 1:**
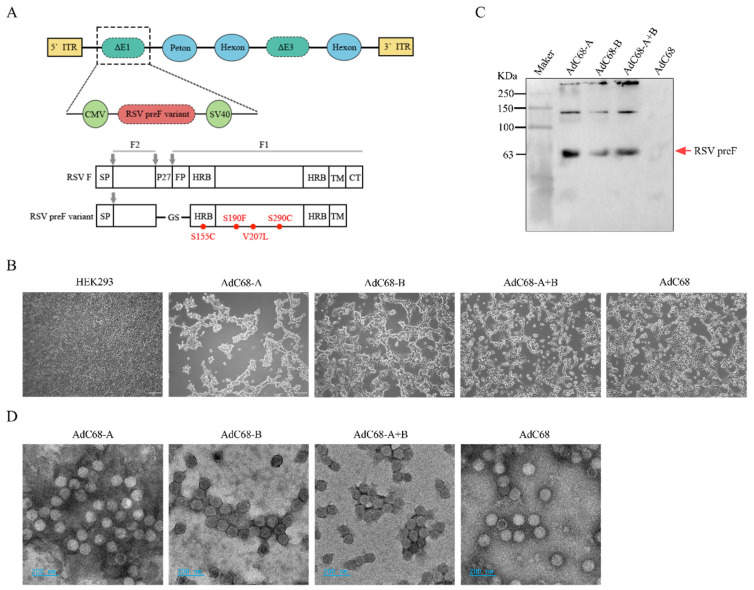
Construction and characterization of RSV pre-F adenoviral vaccine candidates. (**A**) Schematic architecture of recombinant AdC68 plasmids. The codon-optimized RSV pre-F gene (RSV-A, RSV-B, or dual expression cassette) was inserted into the E1-deleted region under transcriptional control of the CMV promoter (CMV p) and simian virus 40 polyadenylation signal (SV40 pA). Critical structural domains are annotated: Furin cleavage site (FC); signal peptide (SP); p27 peptide (removed post-cleavage); fusion peptide (FP); heptad repeat domains A/B (HRA/HRB); transmembrane domain (TM); cytoplasmic tail (CT). (**B**) Cytopathic effects (CPEs) in HEK293 cells were captured 24 h post-infection. Scale bar: 100 µm. (**C**) Western blot analysis under non-reducing/non-denaturing conditions detects RSV pre-F protein oligomeric states in supernatants from HEK293 cells infected with AdC68-A, AdC68-B, AdC68-A+B, or empty vector control (AdC68). Molecular weights are indicated, with bands corresponding to monomeric (63 kDa), dimeric (~126 kDa), and polymeric conformations. (**D**) Negative-stain electron micrographs of purified viral particles (2% uranyl acetate staining). Intact adenoviral capsids (~90 nm diameter) demonstrate preserved structural integrity across all constructs. Scale bar: 200 nm.

The codon-optimized sequence encoding the stabilized prefusion conformation (pre-F) of RSV-A/B fusion (F) glycoprotein was cloned into the E1-deletion site of the AdC68 genome (Figure 1A). Three recombinant chimpanzee adenoviral vaccine plasmids were successfully generated: pAdC68-A expressing RSV-A Pre-F, pAdC68-B expressing RSV-B Pre-F, and pAdC68-A+B co-expressing both RSV-A and RSV-B pre-F proteins. In the AdC68-A+B construct, the RSV-A and RSV-B pre-F sequences were linked via a self-cleaving 2A peptide (ribosomal skipping element) to ensure coordinated expression.

Following plasmid linearization, HEK293 cells were transfected with pAdC68-A, pAdC68-B, or pAdC68-A+B. Viral propagation was monitored through cytopathic effect (CPE) development, with harvest performed when >90% of the monolayer exhibited CPE (Figure 1B). Passage 0 (P0) viral stocks demonstrated characteristic adenoviral aggregation and cell detachment phenotypes. Western blot analysis of infected cell lysates confirmed specific expression of RSV pre-F proteins in all constructs, with no immunoreactive bands detected in empty vector controls (Figure 1C). The 63 kDa monomeric pre-F protein was identified alongside higher molecular weight species corresponding to dimeric and polymeric forms, consistent with proper post-translational processing.

Large-scale production and purification yielded recombinant adenoviral vaccines with the following titers: AdC68-A (1.65 × 10^13^ viral particles [VP]/mL), AdC68-B (2.9 × 10^13^ VP/mL), and AdC68-A+B (2.07 × 10^13^ VP/mL). The control vector AdC68 was prepared at 1.27 × 10^13^ VP/mL. Negative-stain electron microscopy confirmed intact viral particles with characteristic adenoviral morphology (90–100 nm icosahedral capsids) across all constructs, showing no structural differences compared to empty vector controls (Figure 1D).

These results demonstrate the successful rescue of replication-competent chimpanzee adenoviral vectors capable of expressing antigenically authentic RSV pre-F glycoproteins, establishing a foundation for subsequent immunogenicity evaluations.

### 3.2. Single Intranasal Administration of Recombinant AdC68-RSV Vaccines Elicits Robust Cellular Immunity in Mice

**Figure 2:**
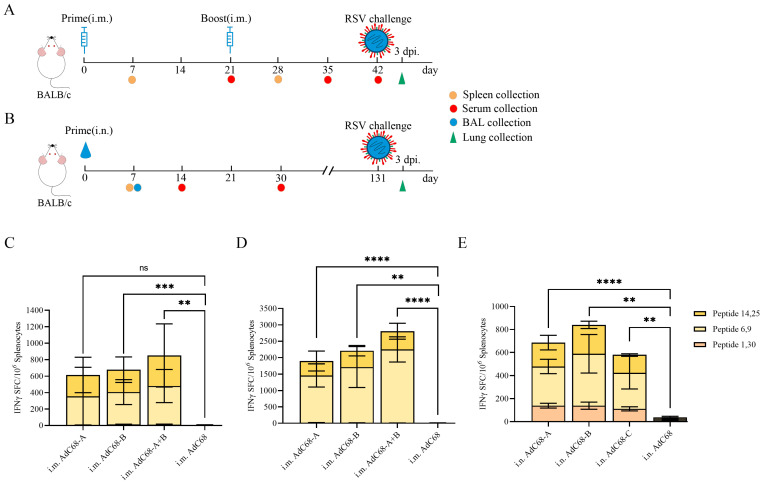
Immunization-challenge schema and vaccine-induced cellular immunity in BALB/c mice. (**A**) Mice (*n* = 13/group) received prime immunization (day 0) with 1 × 10^10^ viral particles (VPs) of AdC68-A, -B, -A+B, or empty vector control (AdC68). Prime–boost groups received homologous chimpanzee adenoviral vaccines at matching doses (prime–boost regimen: days 0/21). (**B**) Mice (*n* = 9/group) were administered 1 × 10^10^ VP via i.n. route (day 0). Control groups received AdC68. Biological samples (spleen, serum, bronchoalveolar lavage [BAL]) were collected at designated intervals. Three days post-challenge, lungs were harvested for viral load quantification. (**C**–**E**) Splenocytes from (**C**,**D**) i.m. and (**E**) i.n. immunized mice were stimulated with three peptide pools (P1,6,9; P14,25; P30) spanning RSV F protein domains. IFN-γ spot-forming cells (SFCs) were quantified per 1 × 10^6^ splenocytes following (**C**) prime and (**D**) boost immunizations. Individual data points represent biological replicates (*n* = 3–4/group), with horizontal bars indicating mean ± SD. ****, *p* < 0.0001; ***, *p* < 0.001; **, *p* < 0.01; ns, *p* ≥ 0.05.

Female BALB/c mice (4–6 weeks old) were immunized via intramuscular (i.m.) or intranasal (i.n.) routes according to the schedules in Figure 2A (i.m.) and 2B (i.n.). Cohorts of four mice (i.m.) and three mice (i.n.) per group were euthanized 7 days post-vaccination for splenocyte isolation. IFN-γ-secreting T cell responses were quantified by ELISpot following stimulation with three RSV F-specific peptide pools.

All vaccine constructs (AdC68-A, AdC68-B, AdC68-A+B) induced significant IFN-γ+ T cell responses compared to empty vector controls (AdC68) across both delivery routes after prime immunization (Figure 2C–E). While intramuscular AdC68-A prime immunization showed comparable responses to controls (*p* > 0.05), suggesting route-dependent efficacy, no significant differences in primary responses were observed between i.m. and i.n. routes (Figure S1A) after a single immunization.

Enhanced T cell reactivity was evident post-boost, with all vaccine groups demonstrating 3.09- to 3.30-fold increases in IFN-γ+ spot-forming cells (SFCs) compared to prime immunization (*p* < 0.01; Figure 2D vs. Figure S1B). Notably, intranasal delivery induced broad peptide pool reactivity, with peptides 1 and 30 preferentially activating i.n.-immunized splenocytes (Figure 2E). This contrasted with the focused epitope recognition pattern observed in i.m. groups, highlighting route-specific immune activation profiles.

### 3.3. Single Intranasal Administration of Recombinant AdC68-RSV Vaccines Induces Antigen-Specific Humoral Responses in Mice

**Figure 3:**
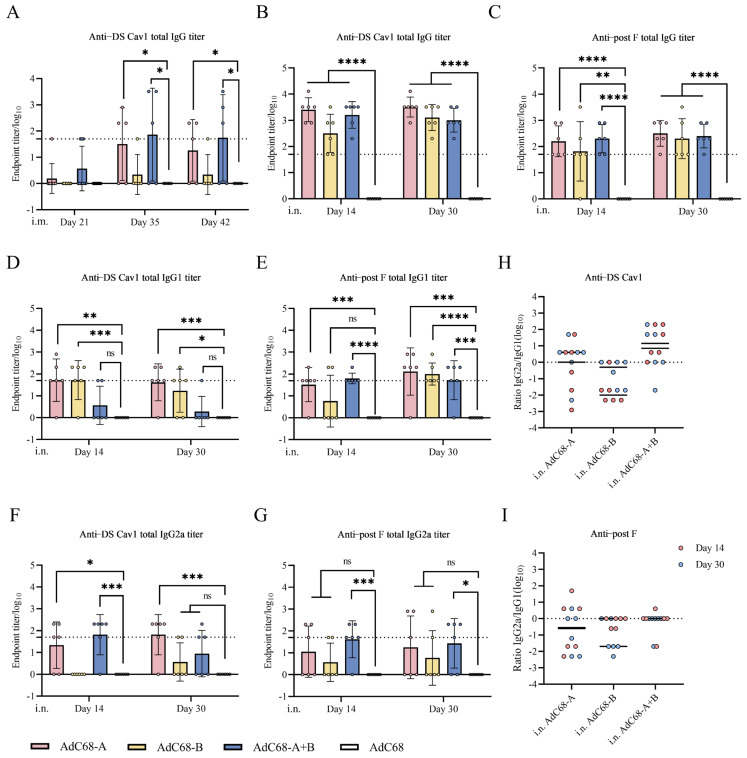
Antigen-specific antibody profiles in immunized mice. (**A**) Intramuscular (i.m.) immunization: Longitudinal analysis of DS Cav1 (pre-F)-specific serum IgG endpoint titers by ELISA at days 21, 35, and 42 post-prime vaccination. (**B**–**G**) Intranasal (i.n.) immunization: Antigen-specific (**B**,**C**) IgG, (**D**,**E**) IgG1, and (**F**,**G**) IgG2a endpoint titers against DS Cav1 (left panels) and post-F (right panels) at days 14/30 post-vaccination. (**H**,**I**) Th polarization indices: IgG2a/IgG1 ratio analysis for (**H**) DS Cav1 and (**I**) post-F responses. Dashed lines indicate assay detection limits (**A**–**G**) or neutral Th1/Th2 balance (**H**,**I**). Individual symbols represent biological replicates (*n* = 5–6/group), with horizontal bars denoting mean ± SD. ****, *p* < 0.0001; ***, *p* < 0.001; **, *p* < 0.01; *, *p* < 0.05; ns, *p* ≥ 0.05.

Primary i.m. immunization with AdC68-A, AdC68-B, or AdC68-A+B elicited low serum IgG titers against DS Cav1 (pre-F conformation). Booster immunization significantly enhanced IgG responses in AdC68-A (*p* = 0.0424) and AdC68-A+B (*p* = 0.0465) groups compared to controls, while AdC68-B showed no significant induction (*p* > 0.05; Figure 3A). IgG levels plateaued between days 35 and 42 post-immunization across all groups, with no detectable responses against post-F protein in i.m.-immunized mice.

The i.n. administration induced distinct humoral profiles, with serum IgG targeting both DS Cav1 and post-F detectable in all vaccine groups by day 14 (Figure 3B,C). Controls remained seronegative throughout the study. Similar to i.m. results, IgG titers showed no significant elevation between days 14–30 post-i.n. immunization. Subclass analysis revealed transient IgG1/IgG2a responses at day 14 (Figure 3D–G), with isotype ratios indicating differential Th polarization: AdC68-A induced balanced Th1/Th2 responses against DS Cav1 in Figure 3H, whereas AdC68-B skewed toward Th2 and AdC68-A+B toward Th1. For post-F antigen, both AdC68-A and AdC68-B exhibited Th2 bias, while AdC68-A+B maintained balanced reactivity in Figure 3I.

Bronchoalveolar lavage (BAL) analysis 7 days post-i.n. immunization revealed undetectable mucosal IgA levels (Table S1), indicating that the elicited humoral responses were predominantly systemic rather than mucosal in nature. Concurrently, serum neutralization assays using RSV long strain showed no measurable neutralizing antibodies across all groups (Figure S2). These collective findings suggest that the humoral immunity generated through our vaccination strategy appears to be principally mediated through non-neutralizing antibody mechanisms.

### 3.4. Single Intranasal Administration of AdC68-RSV Vaccines Confers Prolonged Protection Against Pulmonary RSV Replication in BALB/c Mice

Vaccine efficacy was assessed through RSV challenge at extended time points post-immunization: 42 days for intramuscular (i.m.) and 131 days for intranasal (i.n.) routes. Mice were intranasally infected with 1 × 10^6^ PFU of RSV long strain, with daily weight monitoring and terminal lung viral load quantification via quantitative RT-PCR at day 3 post-challenge.

**Figure 4:**
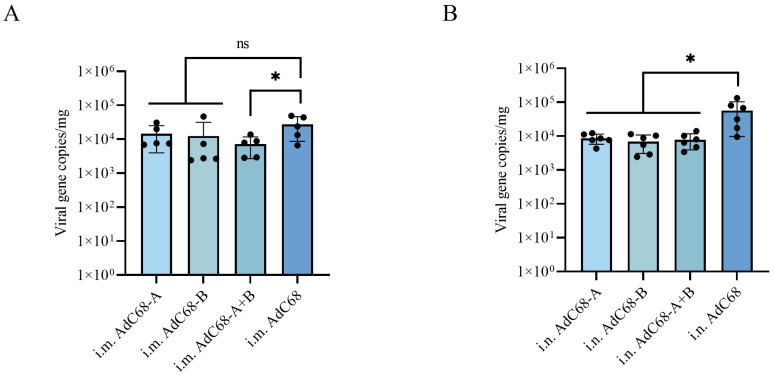
Pulmonary viral load quantification post-RSV challenge. (**A**) Intramuscular (i.m.) and (**B**) intranasal (i.n.) immunized mice: Viral RNA loads in lung homogenates 3 days post-challenge with 1 × 10^6^ PFU RSV long strain, quantified by quantitative RT-PCR (qRT-PCR). RSV genomic copies determined by a standard curve, expressed as copies/mg RNA. Individual symbols represent biological replicates (*n* = 5–6/group), and horizontal bars denote mean ± SD. *, *p* < 0.05; ns, *p* ≥ 0.05.

While residual viral RNA was detected across all groups, distinct protection profiles emerged between immunization routes. i.m. administration of AdC68-A+B significantly reduced pulmonary viral loads compared to controls (73% reduction, *p* = 0.0471; Figure 4A), whereas AdC68-A and AdC68-B showed non-significant attenuation (47%- and 55% reductions, *p* > 0.05). In contrast, i.n. immunization with all vaccine constructs (AdC68-A, -B, -A+B) conferred robust protection, achieving 85%- to 88% reductions in lung viral loads versus controls (*p* < 0.05; Figure 4B). Notably, no significant weight fluctuations were observed during the infection window, suggesting clinical protection despite persistent subclinical viral replication.

## 4. Discussion

The recent FDA approval of three RSV vaccines after six decades of research marks a pivotal advancement [35], yet the continued development of adenoviral vector-based vaccines reflects persistent needs for improved immunization strategies. Building on the proven capacity of replication-deficient ChAd vectors to safely induce potent T/B-cell responses [13,36,37,38,39], we engineered an RSV vaccine candidate using ChAdC68 with three structural modifications: DS-Cav1 stabilizing mutations, P27 peptide/furin cleavage site deletions (replaced by GS linker), and cytoplasmic domain truncation—modifications designed to preserve ø/V neutralizing epitopes while enhancing antigen stability.

Immunization studies demonstrated route-dependent immunological outcomes. Both i.n. and i.m prime immunization elicited comparable cellular responses by day 7 (*p* > 0.05), with i.m. boosting significantly amplifying cellular immunity (3-fold increase vs. prime, *p* < 0.01). Humoral analysis revealed DS-Cav1-specific IgG dominance over post-F IgG (GMT 2383 vs. 422) at day 14 post-i.n. immunization, while i.m. boosting selectively enhanced DS-Cav1 IgG in AdC68-A (GMT 240) and AdC68-A+B (GMT 1320) groups. Notably, serum neutralizing antibodies remained below detection thresholds (<20 ND50) despite antigen-specific IgG induction—a finding paralleling observations in PanAd3-RSV studies where mucosal IgA was transiently detected [19,33,40], contrasting with our undetectable BAL IgA at day 7 post-i.n. administration. Comparative data from Ad26.RSV constructs further validated pre-F antigen superiority, with Ad26.RSV. preF demonstrating 68.2% clinical efficacy [36,37] versus limited protection from wild-type F variants [34,35].

Critical safety assessments addressed historical FI-RSV vaccine failures associated with pathogenic Th2 bias (IgG1/IgG2a > 15) [17,41,42,43,44,45,46]. Our Th1/Th2 profiling through IgG1/IgG2a ratios revealed distinct polarization patterns: AdC68-A exhibited balanced Th2 bias, AdC68-B showed pronounced Th2 skewing, and AdC68-A+B demonstrated Th1 predominance. These profiles, substantially divergent from FI-RSV’s risk-associated ratios, suggest mitigated safety concerns. Intramuscular groups were excluded from the Th analysis due to suboptimal IgG titers (GMT < 40), underscoring the necessity for route-specific immune response characterization.

The 7-day post-intranasal (i.n.) immunization timepoint for immune monitoring was selected based on murine studies demonstrating peak nasal IgA production within this window [47], allowing assessment of primary mucosal engagement. While mucosal IgA and serum (7, 14, and 30 days after the immunization) neutralizing antibodies remained below detection thresholds, the durable protection observed at day 131 likely reflects the differentiation of tissue-resident memory T cells (TRMs) and/or memory B cell pools. Notably, intranasal vaccine-mediated protection against respiratory viruses can operate independently of serum-neutralizing antibodies, as exemplified by TRM populations that provide frontline defense at mucosal portals [48]. These non-circulating effectors rapidly produce antiviral cytokines (e.g., IFN-γ, TNF-α) and recruit secondary immune cells upon pathogen encounter. Furthermore, mucosal plasma cells maintain a quiescent state under homeostasis, with baseline IgA secretion potentially below assay sensitivity yet capable of rapid secretory amplification during infection [49]. We acknowledge that comprehensive pre-challenge immune profiling would strengthen temporal correlation analyses. However, our post-exposure virological assessments provide functional validation of pulmonary protection. Future studies incorporating longitudinal immune monitoring and TRM-specific phenotyping will further delineate the mechanistic hierarchy between humoral and cellular memory components.

The potential impact of vector-specific immune responses warrants consideration in the context of prime–boost vaccination regimens. A key question arises: could anti-vector antibodies (Abs) contribute to diminished IgG responses following booster immunization? Some studies indicate that AdC68-specific neutralizing antibodies (NAbs) remained at baseline levels after primary immunization but exhibited a significant elevation following booster administration [50]. Further analysis of pre-existing adenovirus immunity demonstrated that prior exposure to AdC68 or AdHu5 vectors did not impair the induction of RBD/HA-specific IgG antibodies in immunized mice [50]. These findings collectively suggest that humoral immune responses to the target antigen remain unimpaired by pre-existing adenovirus immunity, supporting the feasibility of adenovirus-based prime–boost strategies.

## 5. Conclusions

Our study establishes that intranasal AdC68-RSV vaccination provides durable pulmonary protection against RSV in BALB/c mice, mediated through robust cellular immunity and antigen-specific IgG responses despite undetectable serum-neutralizing antibodies. While these findings highlight non-neutralizing antibody-dependent protective mechanisms, further mechanistic studies and comprehensive evaluations incorporating pathological assessments are required to fully characterize vaccine efficacy.

## Data Availability

The data generated in the present study may be requested from the corresponding author.

## Supplementary Materials

The following supporting information can be downloaded at https://www.mdpi.com/article/10.3390/vaccines13050528/s1, Figure S1: IFN-γ secreted T-cells induced by recombinant AdC68 vaccination. Figure S2. Representative plaque reduction experiment results showing serum-neutralizing antibodies below detection thresholds. Figure S3: Uncropped and unprocessed images used to generate Figure 1C. Table S1: OD450 values obtained from DS-Cav1-specific IgA detection using ELISA.
