# Supplementary material for: Single-Dose Intranasal Immunization with ChAd68-Vectored Prefusion F Vaccines Confers Sustained Protection Against Respiratory Syncytial Virus in Murine Models
Source: Vaccines (Basel). 2025 May 15;13(5):528. doi: 10.3390/vaccines13050528 (PMC12115431; doi:10.3390/vaccines13050528)
Supplement: Supplementary file 1 [file vaccines-13-00528-s001.zip › vaccines-3577328-supplementary.pdf]

Supplementary Information

Single-Dose Intranasal Immunization with ChAd68-Vectored Prefusion F Vaccines Confers Sustained Protection Against Respiratory Syncytial Virus in Murine Models

Jing Miao[1,2], Xuejie Li[1,2], Yingwen Li[1], Lingjing Mao[1,2], Wenkai Suo[1,2], Jiaming Lan[1,*]

1 Shanghai Institute of Immunity and Infection, Chinese Academy of Sciences, Shanghai 200031, China

2 University of Chinese Academy of Sciences, Beijing 100049, China

* Correspondence: jmlan@ips.ac.cn; Tel.: +86-021-54923061

[*]Corresponding author

Jiaming Lan, Ph.D (e-mail: jmlan@ips.ac.cn)

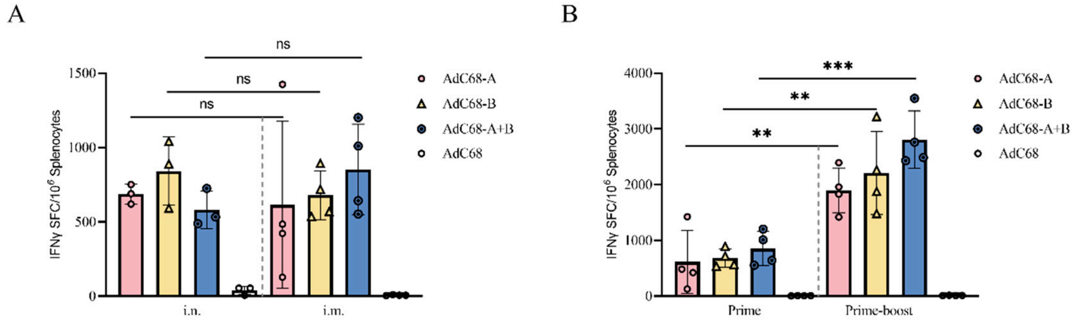

Figure S1. IFN-γ secreted T-cells induced by recombinant AdC68 vaccination. (A) Comparisons of SFCs per 10^6 splenocytes after prime vaccinations were made between i.n. route and i.m. route. (B) Comparisons of SFCs per 10^6 splenocytes were made between the prime and boost vaccinations via i.m. route. The symbols represent the individual animals in the groups.

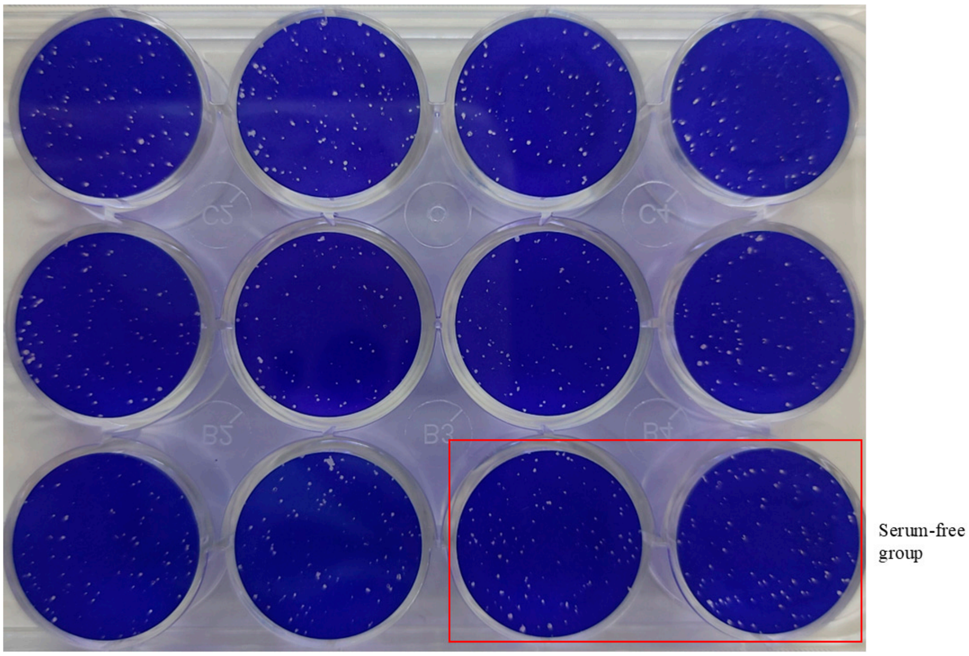

Figure S2. Representative plaque reduction experiment results showing serum neutralizing antibodies below detection thresholds. Serum-free group contained in the red box.

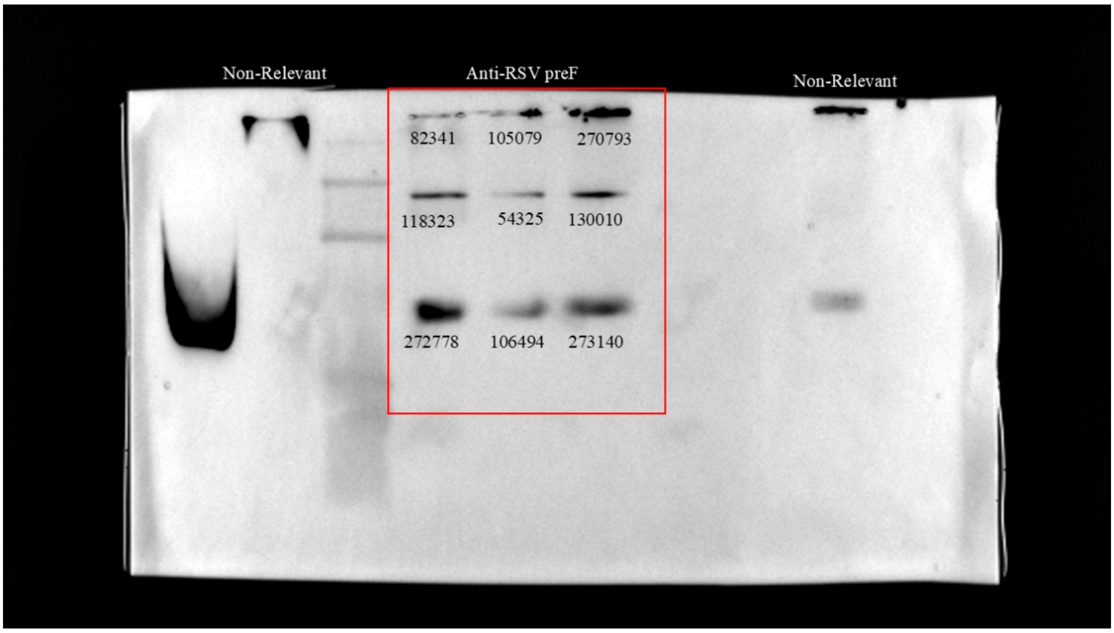

Figure S3. Un-cropped and unprocessed images used to generate Figure 1C. Red boxes indicate the regions shown in Figure 1C. The numbers are the IntDen of the corresponding bans. Other bands are not relevant to this paper.

Table S1. OD450 values obtained from DS-Cav1 specific IgA detection using ELISA.

Sample name	Dilution	OD450
AdC68-A-1	1	0.065
AdC68-A-2	1	0.05
AdC68-A-3	1	0.051
AdC68-B-1	1	0.057
AdC68-B-2	1	0.049
AdC68-B-3	1	0.054
AdC68-A+B-1	1	0.061
AdC68-A+B-2	1	0.056
AdC68-A+B-3	1	0.052
AdC68-1	1	0.047
AdC68-2	1	0.064
AdC68-3	1	0.056
AdC68-A-1	50	0.049

AdC68-A-2	50	0.046
AdC68-A-3	50	0.044
AdC68-B-1	50	0.046
AdC68-B-2	50	0.052
AdC68-B-3	50	0.045
AdC68-A+B-1	50	0.048
AdC68-A+B-2	50	0.045
AdC68-A+B-3	50	0.044
AdC68-1	50	0.045
AdC68-2	50	0.051
AdC68-3	50	0.048
Blank-1	-	0.041
Blank-2	-	0.045
Blank-3	-	0.041
Blank-4	-	0.043
Blank-5	-	0.042
Blank-6	-	0.043
Blank-7	-	0.043
Blank-8	-	0.046
Blank-9	-	0.042
Blank-10	-	0.041
Blank-11	-	0.041
Blank-12	-	0.042
